# Prevention of bacterial colonization on non-thermal atmospheric plasma treated surgical sutures for control and prevention of surgical site infections

**DOI:** 10.1371/journal.pone.0202703

**Published:** 2018-09-05

**Authors:** Utku Kürşat Ercan, Fatma İbiş, Caner Dikyol, Nesrin Horzum, Ozan Karaman, Çağla Yıldırım, Elif Çukur, Emine Afra Demirci

**Affiliations:** 1 Department of Biomedical Engineering, Faculty of Engineering and Architecture, İzmir Katip Çelebi University, Çiğli, İzmir, Turkey; 2 Department of Engineering Sciences, Faculty of Engineering and Architecture, İzmir Katip Çelebi University, Çiğli, İzmir, Turkey; Kwangwoon University, REPUBLIC OF KOREA

## Abstract

Surgical site infections have a remarkable impact on morbidity, extended hospitalization and mortality. Sutures strongly contribute to development of surgical site infections as they are considered foreign material in the human body. Sutures serve as excellent surfaces for microbial adherence and subsequent colonization, biofilm formation and infection on the site of a surgery. Various antimicrobial sutures have been developed to prevent suture-mediated surgical site infection. However, depending on the site of surgery, antimicrobial sutures may remain ineffective, and antimicrobial agents on them might have drawbacks. Plasma, defined as the fourth state of matter, composed of ionized gas, reactive oxygen and nitrogen species, free radical and neutrals, draws attention for the control and prevention of hospital-acquired infections due to its excellent antimicrobial activities. In the present study, the efficacy of non-thermal atmospheric plasma treatment for prevention of surgical site infections was investigated. First, contaminated poly (glycolic-co-lactic acid), polyglycolic acid, polydioxanone and poly (glycolic acid-co-caprolactone) sutures were treated with non-thermal atmospheric plasma to eradicate contaminating bacteria like *Staphylococcus aureus* and *Escherichia coli*. Moreover, sutures were pre-treated with non-thermal atmospheric plasma and then exposed to *S*. *aureus* and *E*. *coli*. Our results revealed that non-thermal atmospheric plasma treatment effectively eradicates contaminating bacteria on sutures, and non-thermal atmospheric plasma pre-treatment effectively prevents bacterial colonization on sutures without altering their mechanical properties. Chemical characterization of sutures was performed with FT-IR and XPS and results showed that non-thermal atmospheric plasma treatment substantially increased the hydrophilicity of sutures which might be the primary mechanism for the prevention of bacterial colonization. In conclusion, plasma-treated sutures could be considered as novel alternative materials for the control and prevention of surgical site infections.

## Introduction

Surgical site infections (SSIs) are the most common among hospital-acquired infections (HAI) and their incidence may rise up to 25% depending on the anatomical location of the surgery site [1]. SSIs not only increase mortality and morbidity rates, but also have a remarkable impact on healthcare costs, bringing an additional 9.7 days of hospitalization and about $25.000 of additional economic burden per patient [2–4].

Despite the rapidly and vastly increasing medical technologies, surgeries are still an inevitable part of modern medicine. Despite the introduction of novel surgical incision closure methods such as surgical staples, sutures are still the most common method for the closure of surgical incisions, ever since their first use which dates back to 3500 BC and form an invariable part of surgical procedures [5–8]. A surgical suture is a filament-shaped medical device that holds the open ends of a wound together and must withstand to normal levels of physiological mechanical stress, in order to provide sufficient mechanical support for wound closure [8]. Sutures can be classified into absorbable or non-absorbable categories based on their biodegradability, and as monofilament or multifilament (or braided) depending on their thread type [9].

Sutures play a crucial role in the closure of surgical wounds. However, similar to other medical devices that are implanted in the body, sutures are foreign bodies on the wound site and act as a nidus for bacterial attachment. Therefore, the presence of sutures significantly increases the susceptibility of a wound to infections, and subsequently increases the risk of an infection on a surgical site [10, 11]. The presence of sutures on a wound site increases the risk of infection by about 10.000 times [12]. While the number of bacteria that are capable of causing an infection on an open wound is about 10^5^ colony-forming unit (CFU) per gram tissue, this number drastically drops to about 10^2^ CFU/per gram tissue when a suture is used for the closure of a wound [13]. Furthermore, the antibiotic susceptibility of bacteria is greatly reduced when they are colonized on the surface of the suture [11]. Infection on the site of a surgical wound not only jeopardizes the desired wound healing process, but might also evolve into life- threatening conditions, especially in critically ill patients [12, 14]. Similar to other implanted medical device-related infections, biofilm formation is the key process for the pathogenesis of suture-related SSIs [2, 15].

Biofilm formation on surgical sutures is a complex process which starts with the adhesion of pathogens on the suture material. Bacterial adhesion in the first 4–6 hours following the implantation of sutures to the surgical wound is critical for biofilm formation and development of suture-related SSIs consequently [10, 16]. Moreover, initial bacterial adhesion on a foreign body as part of biofilm formation is reported as the most important virulence factors of *E*. *coli* and *S*. *aureus* [17, 18]. Therefore, prevention of initial bacterial adhesion and/or inactivation of readily adhered bacteria shortly after implantation of a surgical suture is considered as a critical attempt for the prevention of suture-related SSIs.

In addition to pathogens’ virulence factors, physical properties and chemical composition of suture materials are interrelatedly effective on bacterial adhesion [19–21].

Triclosan-coated antimicrobial sutures were developed and widely used to prevent bacterial adhesion on the suture surface for the prevention of surgical site infections [12]. Triclosan is a potent antimicrobial agent whose efficacy was shown both on Gram-positive and Gram-negative pathogens [3]. Antimicrobial efficacy and successful clinical performance of triclosan coated sutures for prevention of SSIs were reported by various authors [11, 22]. However, clinical benefits which could be obtained from triclosan-coated sutures are highly dependent on the type and anatomical site of the surgery. While outcomes of the utilization of triclosan-coated sutures in sternal surgery, abdominal wall closure and cerebrospinal fluid shunt operations were satisfying for prevention of SSIs, no significant improvement on the prevention of SSIs following appendicitis, breast cancer and colorectal surgeries were shown [1]. Widespread use of triclosan in antimicrobial sutures and its unnecessary use in soap led to development of bacterial resistance via various mechanisms, including the overexpression of efflux pumps and degradative enzymes [23]. Moreover, bioaccumulation of triclosan in human milk, umbilical cord blood, fat tissue and urine was reported. Bioaccumulation of triclosan in the human body was linked to possible undesirable effects on the immune, endocrine and reproductive systems [24].

Metallic substances with antimicrobial activity such as silver, have been taken into consideration to develop antimicrobial sutures to combat with SSIs. However, topography dependent prevention of bacterial adhesion, and desirable tissue compatibility with silver have not yet been fully understood and proven [10].

Similarly, chlorhexidine-coated sutures could be an alternative for the prevention of SSIs due to its broad spectrum and efficient antimicrobial activity. However, short lasting antimicrobial effect and restricted biocompatibility of chlorohexidine, may limit clinical outcomes of chlorohexidine coated sutures for prevention of SSIs [1]. Therefore, a novel effective suture capable of preventing the initial adhesion of pathogens to the suture surface for the prevention of suture-related SSIs is still needed.

Plasma is defined as the fourth state of matter and ionized gas. Plasma could be artificially generated by applying an external electric field to a gas. When an external electric field is applied to a volume of gas, free electrons are accelerated under the influence of an applied electric field, to acquire higher kinetic energy. Accelerated free electrons then collide with gas atoms and/or molecules and causes removal of electrons (electrons removed from gas molecules are called secondary electrons) from gas molecules, leading to the ionization of gas and formation of plasma [25, 26]. Depending on the temperature of the gas, plasmas are classified as thermal and non-thermal plasmas. In non-thermal plasmas, free electrons might reach up to several thousand kelvins, while the ambient gas remains in room temperature. Therefore, non-thermal plasmas could be used in biomedical applications and the treatment of heat-sensitive materials [27].

Biomedical effects of non-thermal plasmas were mainly attributed to free radicals, reactive oxygen species (ROS), reactive nitrogen species (RNS), free electrons, ultraviolet (UV) photons and electric field, that are generated during the formation of plasma [28].

Excellent and broad spectrum antimicrobial effects of non-thermal atmospheric plasma (NTAP) on planktonic and biofilm forms of pathogens, including multi-drug resistant strains, are well documented [29–31]. Not only does NTAP show antimicrobial activity by itself, but also materials such as liquids (namely, deionized water, phosphate-buffered saline solution), gels, and ultrasound contrast agents. Even metallic surfaces possess and show antimicrobial activity when treated with NTAP [28, 32–37]. For instance, in one of our previous studies we treated alginate gels with a similar NTAP setup and demonstrated that alginate gels could acquire antimicrobial activity [35]. Similarly, after the NTAP treatment of common implant materials, biofilm formation was remarkably reduced on those materials [37]. In another study, Yorsaeng et al. showed that latex surgical gloves could gain antimicrobial activity by cold plasma treatment [38]. In the present study, to the best of our knowledge for the first time in the literature, NTAP treatment was used to obtain sutures that could be capable of preventing bacterial adhesion for the prevention of suture-related SSIs.

In the present study, two braided multifilament sutures; poly (glycolic-co-lactic acid) (PGLA), (polyglycolic acid) PGA and two monofilament sutures; (polydioxanone) PDO, poly (glycolic acid-co-caprolactone) PGCL were treated with NTAP for the prevention of suture-related SSIs. Activity of NTAP-treated sutures were tested on *Escherichia coli* (*E*. *coli*) and *Staphylococcus aureus* (*S*. *aureus*). Moreover, microbial inactivation efficacy of NTAP was tested on contaminated suture samples to eradicate already adhered pathogens. Furthermore, the effects of NTAP treatment on mechanical, degradation and surface properties of sutures were evaluated. The possible chemical modifications, dependent on NTAP, on sutures were characterized using FTIR and XPS. The influence of NTAP-treated sutures on *in vitro* wound healing was also investigated.

## Materials and methods

### Preparation and plasma treatment of suture samples

In the present study, sterile, absorbable, United States Pharmacopeia (USP) size 1, two braided multifilament sutures; PGLA, PGA and, two monofilament sutures; PDO, PGCL were used. Also, antibacterial PGLA (ALCALACTINE) suture samples, which contain chlorhexidine diacetate as antimicrobial agent, were used as positive control in colonization experiments. All suture samples were generously donated by KATSAN A.S (İzmir, Turkey).

A custom-made dielectric barrier discharge (DBD) electrode was connected to a microsecond pulsed alternating current (AC) power supply to generate non-thermal atmospheric DBD air plasma (Advanced Plasma Solutions, Malvern, PA, USA). The DBD electrode was constructed by covering a 10-mm thick copper plate (38mm x 64 mm) with a 1-mm thick glass slide (50mm x 75mm). The remaining copper plate was placed inside a polyethylene housing to insulate the exposed surfaces. For all experiments, power supply was operated at 32 kV peak-to-peak voltage, 2.5 kHz frequency and 10 μs pulse duration which yields 0.65 W/cm^2^ of power density. The discharge gap was fixed as 1mm.

**Fig 1:**
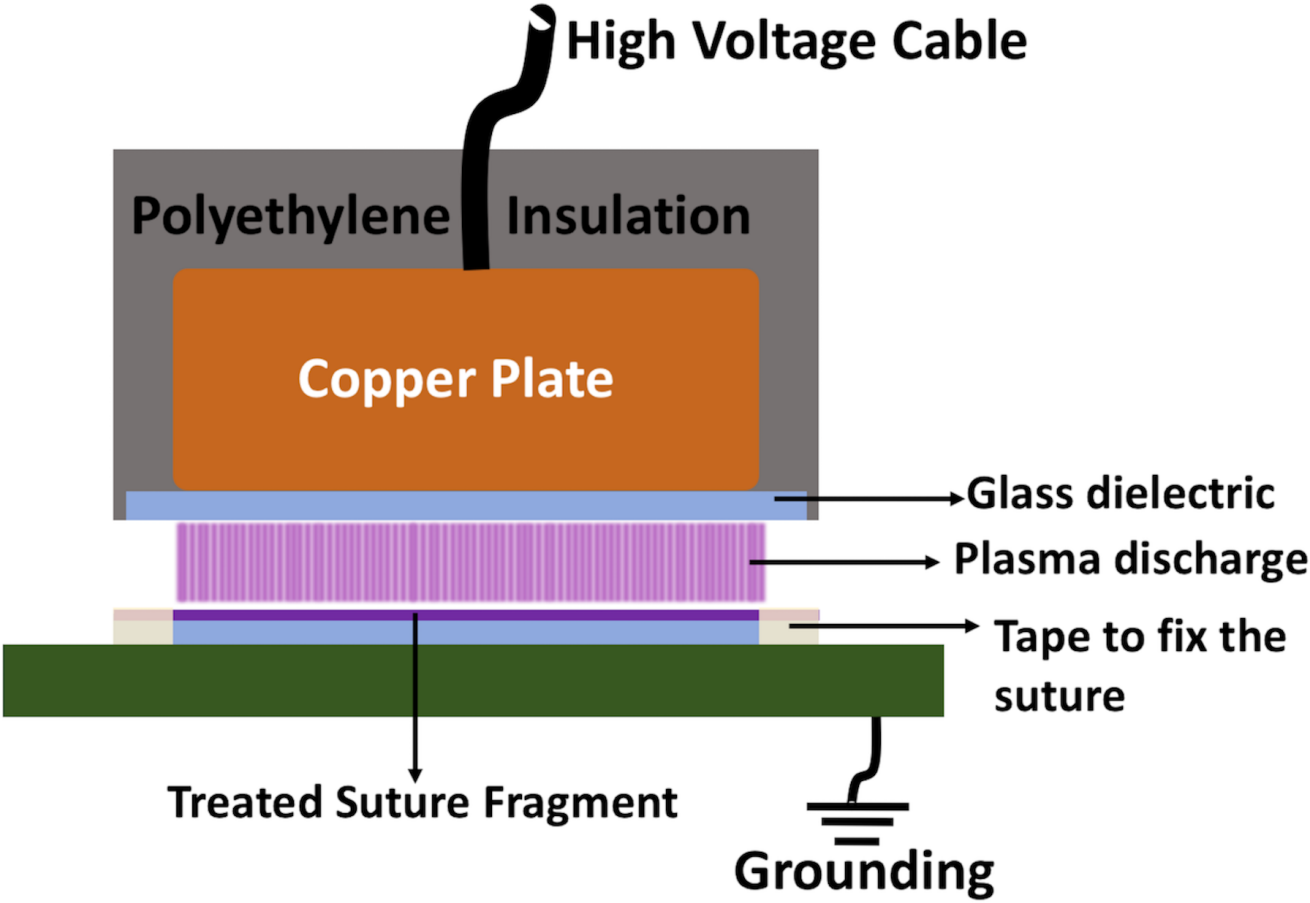
Schematic of NTAP treatment setup of sutures. A schematic that illustrates the NTAP treatment of suture fragments. Note that sutures were fixed to glass slide over the grounding electrode using tapes, and parts of suture fragment remaining under the tape were removed after completion of NTAP treatment.

Approximately 7.5 cm long segments of sutures were cut and fixed on a 1-mm thick glass slide by placing autoclave tape to 7 mm distance at both ends of sutures in order to provide complete exposure of the suture sample to plasma discharge (Fig 1). After the completion of plasma treatment, 5 cm suture fragments that were completely exposed to plasma discharge were removed by scissors. In all experiments, except decontamination tests, samples were treated for 7 minutes. In decontamination experiments, plasma treatments were performed for 3 minutes. In microbiological experiments *E*. *coli* ATCC 25922 and *S*. *aureus* ATCC 25923 were used as gram negative and gram positive model organisms, respectively.

### Decontamination of sutures

In decontamination experiments, sutures were first contaminated separately with *E*. *coli* and *S*. *aureus* by suspending suture fragments in 10^5^ CFU/ml microbial suspensions and incubated for 12 hours at 37°C in a stationary incubator to allow the colonization of pathogens. After incubation, sutures were washed with 1X sterile phosphate buffered saline (PBS) solution for once and exposed to NTAP treatment for 3 minutes at 32 kV peak-to-peak voltage, 2.5 kHz frequency and 10 μs pulse duration which yields 0.65 W/cm^2^ of power density. A three-minute plasma treatment time for the decontamination of sutures, was determined in the light of a previously conducted pilot study with *E*. *coli* on PGLA suture (S1 Fig).

After the plasma treatment, suture samples were placed on trypticase soy agar (TSA) petri dishes and incubated for 24 hours at 37°C in a stationary incubator, to assess microbial growth visually. Moreover, the decontamination efficacy of NTAP was quantified by colony-counting assay. For this purpose, contaminated suture samples were similarly treated with NTAP for 3 minutes, washed with 1X sterile PBS solution in a similar fashion, and then placed in microcentrifuge tubes containing 1X sterile PBS. Microcentrifuge tubes were shaken and vortexed thoroughly to allow the homogenization of pathogens in the PBS solution. Afterwards, serial dilutions were made appropriately and samples were plated on TSA petri dishes. Petri dishes were incubated for 24 hours at 37°C in a stationary incubator and the next day, surviving colonies were counted. Untreated suture samples were used as negative control. Results were enumerated as log10(N) bacteria/cm suture.

### Prevention of bacterial colonization on sutures

Colonization experiments were performed to evaluate the efficacy of NTAP treatment for the prevention of microbial colonization on surgical sutures. As opposed to decontamination experiments, suture samples were first treated with NTAP and then placed in microbial suspension in colonization experiments.

In detail, suture fragments first treated with NTAP for 7 minutes at 32 kV peak-to-peak voltage, 2.5 kHz frequency and 10 μs pulse duration which yields 0.65 W/cm^2^ of power density. A seven-minute plasma treatment time for the prevention of bacterial colonization on sutures was determined in the light of a previously conducted pilot study with *E*. *coli* on PGLA suture (S2 Fig). Afterwards, NTAP-treated suture fragments were placed inside 10^5^ CFU/ml microbial suspensions of *E*. *coli* and *S*. *aureus* separately, and incubated for 12 hours at 37°C in a stationary incubator, to allow the colonization of pathogens. Following the incubation period, suture samples were gently washed with 1X sterile PBS solution to remove non-adherent pathogens, and then placed on TSA petri dishes and incubated for 24 hours at 37°C in a stationary incubator, to assess microbial growth visually.

Moreover, the prevention of bacterial colonization efficacy by NTAP treatment was quantified by colony counting assay. For this purpose, suture samples were similarly treated with NTAP for 7 minutes, and then placed inside 10^5^ CFU/ml microbial suspensions of *E*. *coli* and *S*. *aureus* separately and incubated for 12 hours at 37°C in a stationary incubator, to allow colonization of pathogens. After incubation samples were gently washed with 1X sterile PBS for once and placed in microcentrifuge tubes containing 1X sterile PBS. Microcentrifuge tubes were shaken and vortexed thoroughly to allow homogenization of pathogens in the PBS solution. Afterwards, serial dilutions were made appropriately and samples were plated on TSA petri dishes which were then incubated for 24 hours at 37°C in a stationary incubator. The next day surviving colonies were counted. Untreated suture samples and ALCALATINE sutures were used as negative and positive controls respectively. Results were enumerated as log10(N) bacteria/cm suture.

In addition, the persistency of NTAP treatment, which mediated the prevention of bacterial colonization on sutures, was assessed. Suture samples were prepared and treated with NTAP in a similar fashion as explained. Following the plasma treatment, sutures were kept in sealed, sterile petri dishes at room temperature for 1, 3, 5, 7 and 10 days. After the completion of storage period, sutures were placed in bacterial suspensions and then bacterial colonization on sutures were evaluated visually and quantitatively as described.

### Contact angle measurements

The effect of NTAP treatment on the hydrophilicity of suture samples was analyzed using a goniometer (KSV Attension Theta, Biolin Scientific, Stockholm, Sweden). For contact angle measurements, 3 replicates of each suture material, including NTAP treated and untreated, were prepared by wrapping around a 1-mm thick glass slide, in order to obtain a surface on which the water droplet was placed. Sutures that were wrapped around the glass slide were treated with NTAP for 7 minutes as described. Contact angle measurements were performed shortly after the NTAP treatments. A 4 μl of distilled water droplet was dispensed to three different random locations on untreated and NTAP treated suture samples. Thus, in total, 9 different measurements were performed for each NTAP treated and untreated suture materials. Each water droplet was analyzed by the software that was provided with the goniometer system. The contact angle was given as the mean of all measurements from each suture sample.

### Degradation of sutures

The effect of the NTAP treatment on the degradation properties of sutures were evaluated by degradation test. All types of sutures were cut to 5 cm fragments and treated with NTAP for 7 minutes as described. After the NTAP treatment, the weights of each suture sample were measured using an analytical balance. After weight measurements, the NTAP treated suture samples were placed in 10 ml of 1X sterile PBS solution and incubated at 37°C and 120 revolutions per minute (rpm) in a shaker incubator throughout the degradation tests. The weight of the sutures were measured using an analytical scale every five days until the samples got disintegrated while handling for the weight measurements. After each measurement, the PBS solution was refreshed. Before weight measurements, the suture samples were removed from the 1X PBS solution, and placed on a filter paper to remove excess liquid, and then transferred and held in a desiccator under vacuum, to allow complete drying for 2 hours. After the drying procedure, the suture samples were weighed using an analytical scale and placed back in a fresh 1X sterile PBS solution. Results were represented as percent weight change.

### Tensile testing of sutures

The mechanical properties of suture samples were evaluated using a universal mechanical test machine (Shimadzu AGS-X 50 kN, Kyoto, Japan). Suture samples were treated for 7 minutes as described. Untreated suture samples were used as control. Untreated and NTAP treated suture samples were placed separately in between the tensile arm of the test machine. The tensile test was performed at a cross-head speed of 300 mm/minutes and 5 cm long samples were stretched until breakage occurred and the maximum load was recorded in Newtons (N).

### In vitro scratch wound assay

The effect of the NTAP treated sutures on wound healing was evaluated through *in vitro* scratch wound assay by using L929 mouse fibroblast cell line. Firstly, sutures were treated with the NTAP for 7 minutes as described. Sutures used in the study, either untreated or NTAP treated, were pre-incubated in a 1 ml serum free Dulbecco’s Modified Eagle’s Medium (DMEM) (Sigma-Aldrich, St. Louis, MO, USA) cell culture medium with agitation at 120 rpm for 24 hours. L929 cells were seeded on a 6-well plate at densities of 1x10^5^ cell/ml per well and incubated in DMEM medium supplemented with 10% fetal bovine serum (FBS) (Sigma-Aldrich, Steinheim, Germany), 1% L-glutamine (Genaxxon BioScinece, Ulm, Germany) and 1% penicillin/streptomycin (Genaxxon BioScinece, Ulm, Germany) at 37°C and 5% CO_2_ environment. Cells were kept in exponential phase and used at passage three. When the cells were close to confluence, a scratch was made on each well using a sterile 200 μl pipet tip to mimic wound and shortly after, the wound areas were visualized with an inverted microscope (Olympus CKX41, Tokyo, Japan). Care was taken during the scratching process to ensure that similar size and distance were produced for all samples. Afterwards, the DMEM medium was removed, and all wells were gently washed using 1X sterile PBS to remove the cell debris from the scratch. Serum-free DMEM medium, in which untreated and NTAP treated suture samples were incubated, was transferred on the scratched cells which were incubated at 37°C and 5% CO_2_ environment.

The wound area was visualized with an inverted microscope (Olympus CKX41, Tokyo, Japan) at 1, 2 and 3 days after exposing the cells to serum-free medium from the incubation of suture samples. Also, serum free DMEM, in which no sample was incubated, was used as the background to determine wound healing.

Moreover, the wound healing effect of NTAP treated sutures was quantified using the Image J software on the images collected with inverted microscope. The wound area at day 0 was set as 100% and wound area at day 1,2 and 3 normalized accordingly.

### Scanning electron microscopy (SEM) imaging

Untreated and NTAP treated suture samples were visualized using a Carl Zeiss 300VP scanning electron microscope (SEM) (Zeiss, Ober4kochen, Germany), in order to investigate the possible destructive effects of the NTAP treatment on suture samples. NTAP treatment was performed for 7 minutes as described for experimental groups. SEM images were collected at an accelerating voltage of 5 kV after coating with gold (QUORUM; Q150 RES; East Sussex; United Kingdom) at 20 mA for 60 seconds, which yielded about a 2-nm thick gold coating.

### Fourier transform infrared (FTIR) spectroscopy analysis

Fourier transform infrared (FTIR) spectra of each type of untreated and 7-minute NTAP treated suture samples were obtained using a Thermo Scientific Nicolet iS5 spectrometer, equipped with a spectral resolution of 4 cm^-1^. The instrument was equipped with iD5 attenuated total reflection (ATR) accessory having a diamond crystal, collecting 16 scans in the 400–4000 cm^-1^.

### X-ray photoelectron spectroscopy (XPS) analysis

X-ray photoelectron spectroscopy (XPS) measurement was performed on each type of untreated and 7-minute NTAP treated suture samples using a Thermo Scientific K-Alpha instrument equipped with Al Kα monochromatized radiation at 1486.7 eV X-ray source. A 180° double-focusing hemispherical electron energy analyzer with a 128- multichannel detector system was used. The analysis chamber was pumped to a pressure of 2 × 10^−7^ mbar. Survey spectra and high-resolution spectra were acquired using a constant analyzer with 50 eV pass energies at single point analysis of each sample’s surface area. The X-ray beam size was 300 μm and the detector input angle was 45°. Data were analyzed using Avantage XPS software with Gaussian/Lorentzian peak shapes and a Shirley/Smart type background. Charge referencing was done by setting the lower binding energy C 1s photopeak at 285.0 eV C 1(s) hydrocarbon peak.

### Statistical analysis

All experiments were replicated three times and three different samples were used in each replication (n = 9) unless otherwise stated. SPSS 13.0 was used for statistical analysis. Student *t*-test and one-way analysis of variance (ANOVA) were conducted for pair comparisons and multiple comparisons respectively, and p< 0.05 was considered as significant.

## Results

### Decontamination of sutures

**Fig 2:**
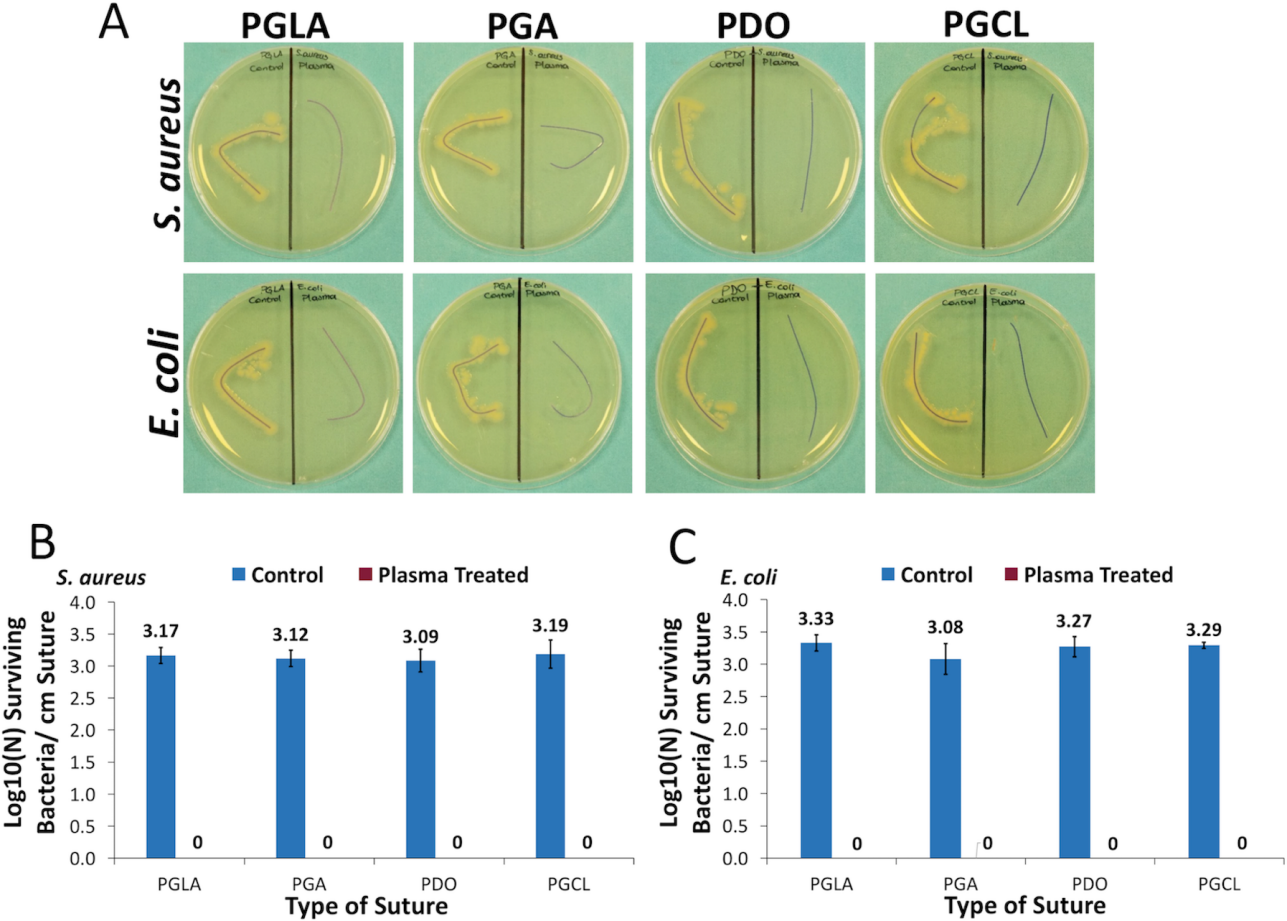
Antimicrobial effect of NTAP treatment on contaminated sutures. (A) Growth of *E*. *coli* and *S*. *aureus* around untreated suture samples was clearly visible on TSA plates while no bacterial growth was observed around 3-minute NTAP treated sutures. Sutures on the left sides of petri dishes are untreated (control suture) and sutures on the right sides of petri dishes are NTAP treated samples. (B) Logarithmic growth of *S*. *aureus* per cm suture fragment of control and 3-minute NTAP treated sutures. (C) Logarithmic growth of *E*. *coli* per cm suture fragment of control and 3-minute NTAP treated sutures.

The colonization of both *E*. *coli* and *S*. *aureus* were determined to be about 3-log for all suture types. 3-minute NTAP treatment of contaminated sutures completely inactivated (p< 0.05) the colonized bacteria on each type of suture samples (Fig 2A–2C). Pictures of contaminated (control) and NTAP-treated suture samples following incubation on TSA plates showed consistent results with colony numbers. Bacterial growth zone was evident around each type of control suture samples for both *E*. *coli* and *S*. *aureus*, while no growth was observed around NTAP-treated suture samples (Fig 2A). Moreover, following further incubation of samples for 48 hours, in order to rule out any possible dormancy, no growth was observed around NTAP treated suture samples for *E*. *coli* and *S*. *aureus*.

### Prevention of bacterial colonization on sutures

**Fig 3:**
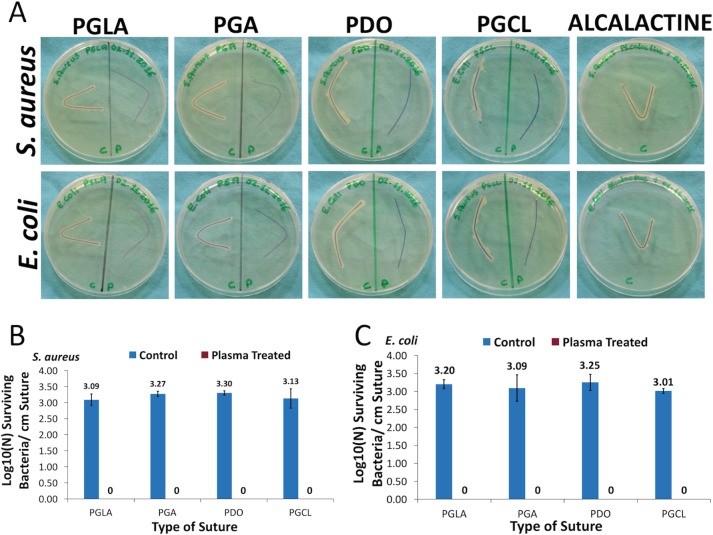
Prevention of bacterial colonization on suture fragments after NTAP treatment. (A) Growth of *S*. *aureus* and *E*. *coli* was observed around the untreated and antimicrobial ALCALACTINE suture samples while no bacterial growth was observed around the 7-minute NTAP treated suture samples on TSA plates. Sutures on the left sides of petri dishes are untreated (control suture) and sutures on the right sides of petri dishes are NTAP treated samples. (B) Logarithmic growth of *S*. *aureus* per cm suture fragment of control and 3-minute NTAP treated sutures. (C) Logarithmic growth of *E*. *coli* per cm suture fragment of control and 3-minute NTAP treated sutures. Note that around 3-log growth of *S*. *aureus* and *E*. *coli* was observed on untreated suture samples while no growth was observed on 7-minute NTAP treated suture samples.

As demonstrated in Fig 3A, 7-minute NTAP treatment, remarkably prevented the colonization of *S*. *aureus* and *E*. *coli* on each type of suture. A growth zone around each type of control sutures that were incubated on TSA plates, was evident, while no growth of *S*. *aureus* and *E*. *coli* was observable around the NTAP-treated sutures. Moreover, a growth zone around the antimicrobial ALCALATINE suture was present. Further incubation of sutures on the TSA plate for 48 hours also didn’t reveal any growth around the NTAP-treated suture samples. These observations were consistent with results represented in Fig 3B and 3C. The colony counting assay results also clearly indicates that the 7-minute NTAP treatment completely prevents the colonization of *S*. *aureus* and *E*. *coli* on each type of NTAP treated suture samples (p< 0.05). Similar to the results of the decontamination experiment, 3-log growth on control sutures was determined, while no growth was determined on NTAP treated sutures.

**Fig 4:**
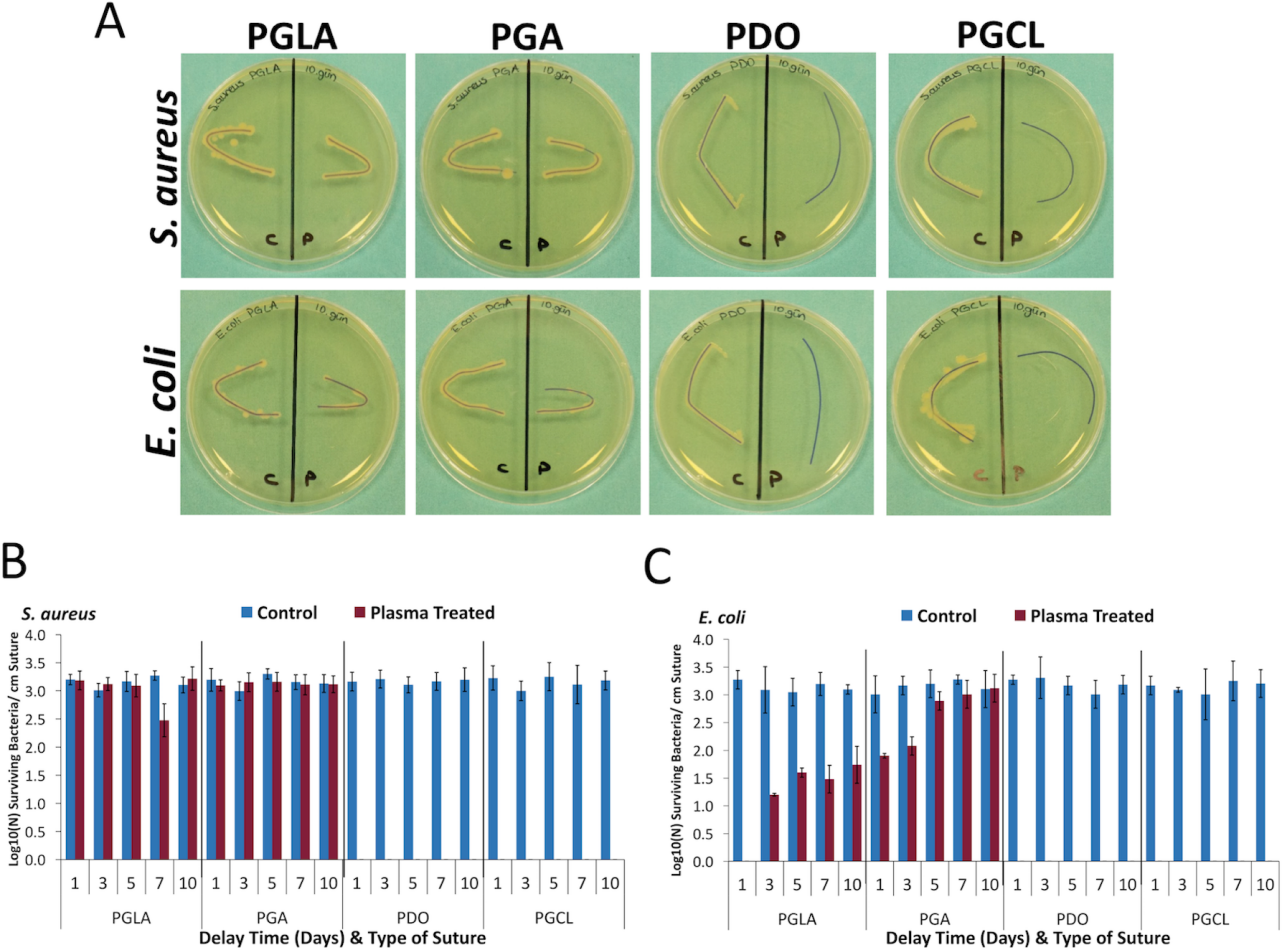
Persistency of bacterial colonization prevention after NTAP treatment. (A) Representative images of growth of *S*. *aureus* and *E*. *coli* on agar plates 10 days after 7-minute NTAP treatment. Note that no growth of *S*. *aureus* and *E*. *coli* was observed PDO and PGCL sutures even after 10 day later NTAP treatment on TSA plates. Sutures on the left sides of petri dishes are untreated (control suture) and sutures on the right sides of petri dishes are NTAP treated samples. (B) Logarithmic growth of *S*. *aureus* on per cm suture fragment 1, 3, 5, 7 and days after 7-minute NTAP treatment. Colonization of *S*. *aureus* on PDO and PGCL sutures was prevented at given time-points, however *S*. *aureus* adhered on PGLA and PGA sutures even one day after 7-minute NTAP treatment. (C) Logarithmic growth of *E*. *coli* on per cm suture fragment 1, 3, 5, 7 and days after 7-minute NTAP treatment. Colonization of *S*. *aureus* on PDO and PGCL sutures was prevented at given time-points, while *S*. *aureus* colonization on PGLA and PGA sutures increases as the delay time increases.

Also, effect of NTAP treatment on the persistent prevention of colonization was assessed for a longer period of time. For this purpose, after completion of the plasma treatment, NTAP-treated sutures were kept for 1, 3, 5, 7, and 10 days in aseptic conditions and room temperature. Afterwards, microbial experiments were carried out. As depicted in Fig 4A, no growth of *S*. *aureus* and *E*. *coli* was observed around the 7-minute NTAP-treated monofilament PDO and PGCL suture samples. However, growth of *S*. *aureus* and *E*. *coli* was visible around the 7-minute NTAP-treated multifilament PGLA and PGA sutures after 10 days of delay. Additionally, as represented in Fig 4B and 4C, colonization of *S*. *aureus* and *E*. *coli* wasn’t detected on the 7-minute NTAP-treated monofilament PDO and PGCL sutures for all the delay time points, whereas around 3-log growth was determined on untreated PDO and PGCL sutures (p< 0.05). However, 3-log bacterial colonization of *S*. *aureus* that is comparable to the untreated control samples, was determined on the 7-minute NTAP-treated multifilament PGLA and PGA sutures for all the delay time points. The colonization prevention effect of the 7-minute NTAP-treated multifilament PGLA and PGA sutures gradually decreased throughout the delay period for *E*. *coli*. *E*. *coli* have shown 3-log colonization on untreated control PGLA and PGA sutures. Colonization of *E*. *coli* on the 7-minute NTAP-treated PGLA sutures after 1 day of delay was not detected, while at day 3, 5, 7, and 10, the colonization of *E*. *coli* was around 1.5 log (p< 0.05). In the 7-minute NTAP-treated PGA sutures, *E*. *coli* colonization showed a gradual increase in which, 1.9, 2.08, 2.89, 3.01 and 3.12 log colonization was observed for 1, 3, 5, 7 and 10 days of delay respectively. Prevention of colonization of *E*. *coli* on the 7-minute NTAP-treated PGA sutures were found to be statistically significant for 1 and 3 days of the delay period (p< 0.05).

### Contact angle measurements

**Fig 5:**
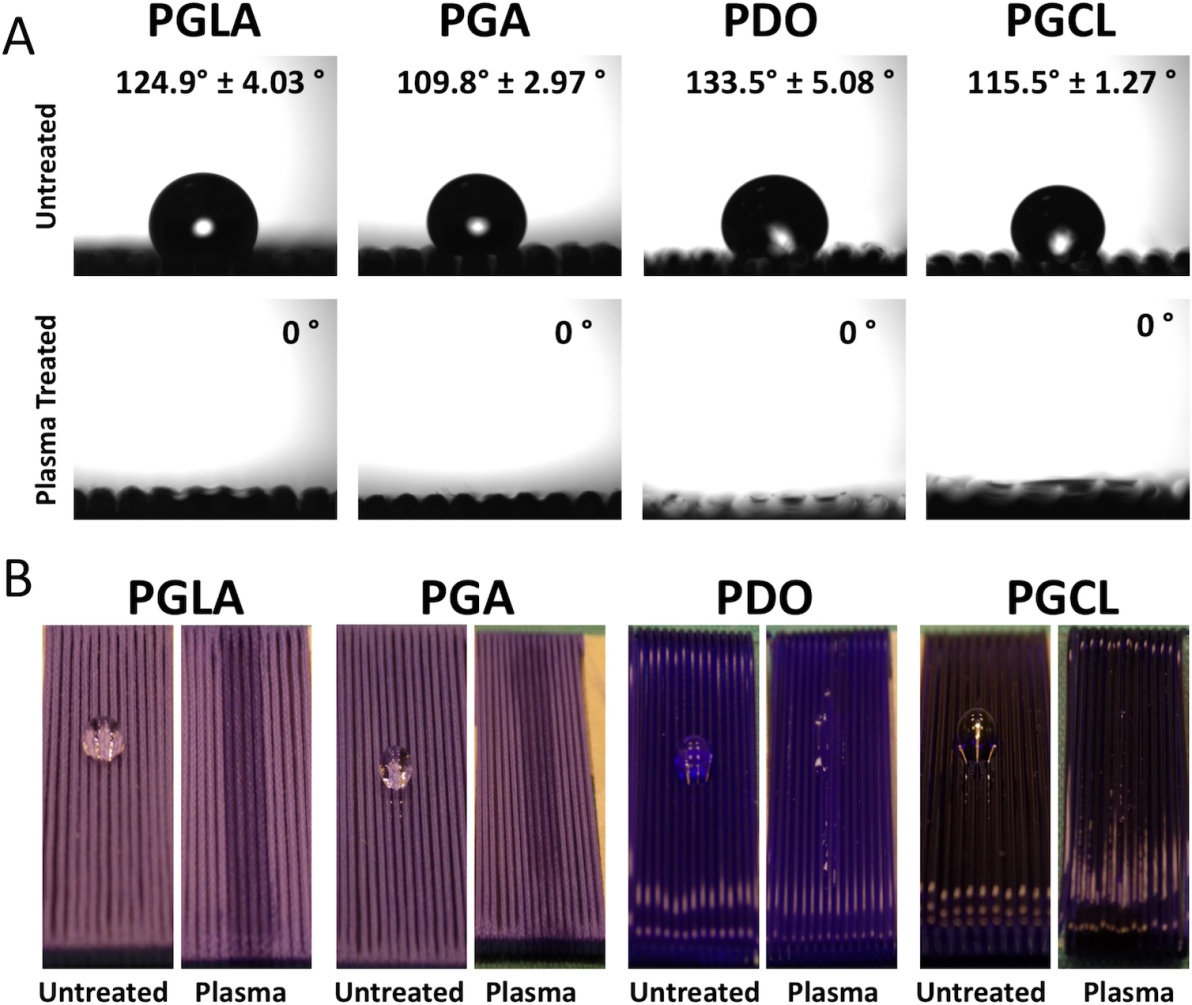
Change in water contact angle on NTAP treated sutures in comparison with untreated sutures. (A) After 7-minute NTAP treatment of sutures, the water contact angle drops to 0° on all suture materials. (B) In consequence of 0° contact angle obtained by 7-minute NTAP treatment, dispersion of water droplet on suture samples could be observed by naked eye.

Contact angle measurements have shown that hydrophilicity of the suture samples remarkably increased after the 7-minute NTAP treatment as shown in Fig 5A. Contact angles of untreated control sutures were determined as 124°, 109°, 133° and 115° for PGLA, PGA, PDO and PGCL, respectively. After the NTAP treatment the contact angles of each suture material were determined as 0° (p< 0.05). Moreover, as shown in Fig 5B, water droplets completely spread over the NTAP-treated sutures.

### Degradation of sutures

**Fig 6:**
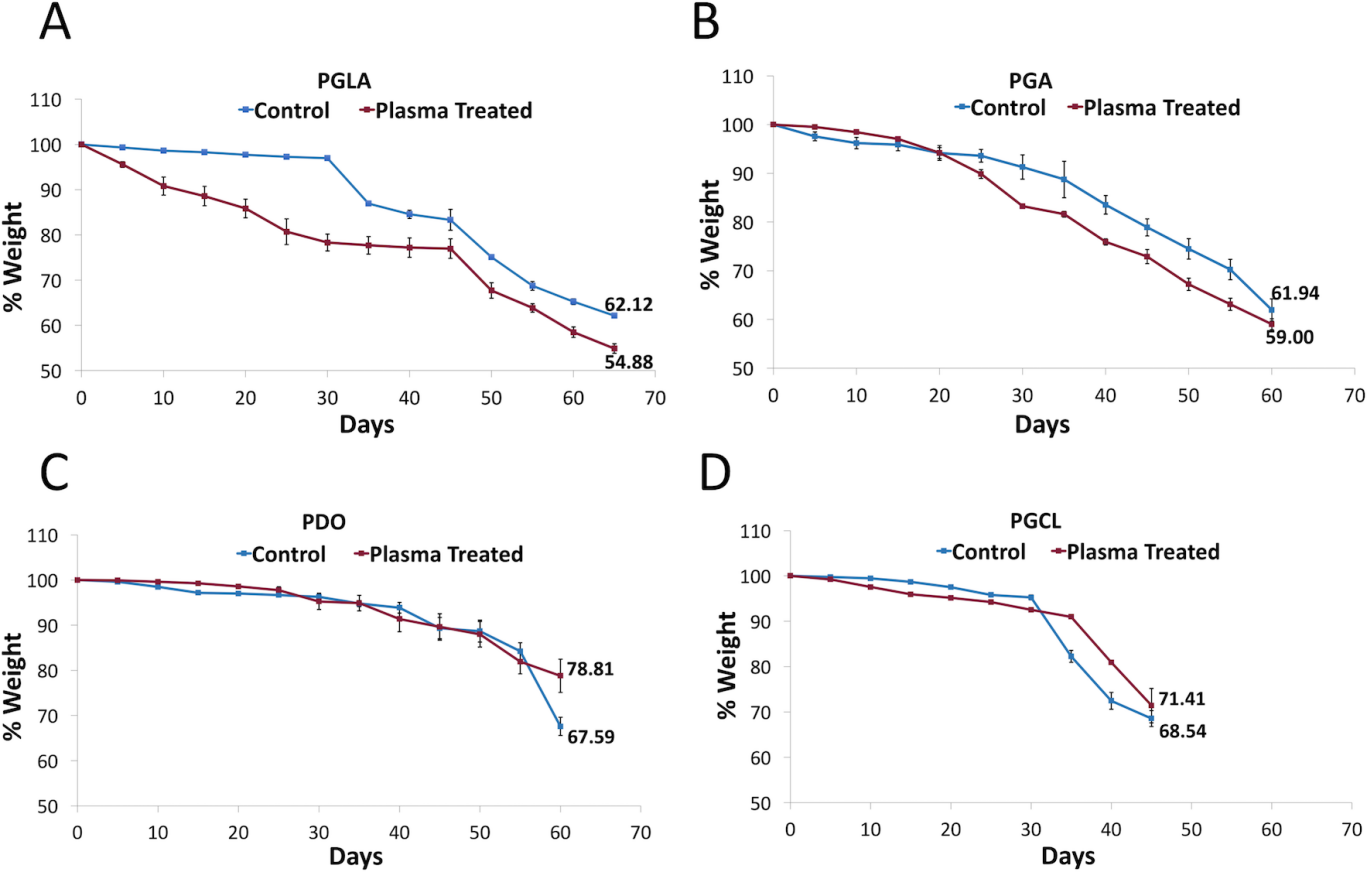
Degradation of suture samples after 7-minute NTAP treatment. Degradation of (A) PGLA, (B) PGA, (C) PDO and (D) PGCL sutures in PBS solution at 37°C was determined by weight measurements until suture fragments got shattered during manipulation.

The weight measurements of control and NTAP-treated sutures were continued until the suture fragments got shattered while manipulating the samples with a tweezer. As represented in Fig 6, the NTAP treatment didn’t changed the time on which sutures got shattered. In detail, the degradation time was determined as 65, 60, 60, and 45 days after the NTAP treatment for both untreated control and the 7-minute NTAP-treated PGLA, PGA, PDO and PGCL sutures respectively. By the time the degradation occurred (in other words, the time at which samples got shattered), the remaining percentage of the weight of the untreated and 7-minute NTAP-treated suture samples were determined as 62% and 54% for PGLA (p< 0.05), 61% and 59% for PGA (p> 0.05), 67% and 78%, for PDO (p< 0.05) and 68% and 71% for PGCL (p> 0.05), respectively (Fig 6A–6D).

### Tensile testing of sutures

**Fig 7:**
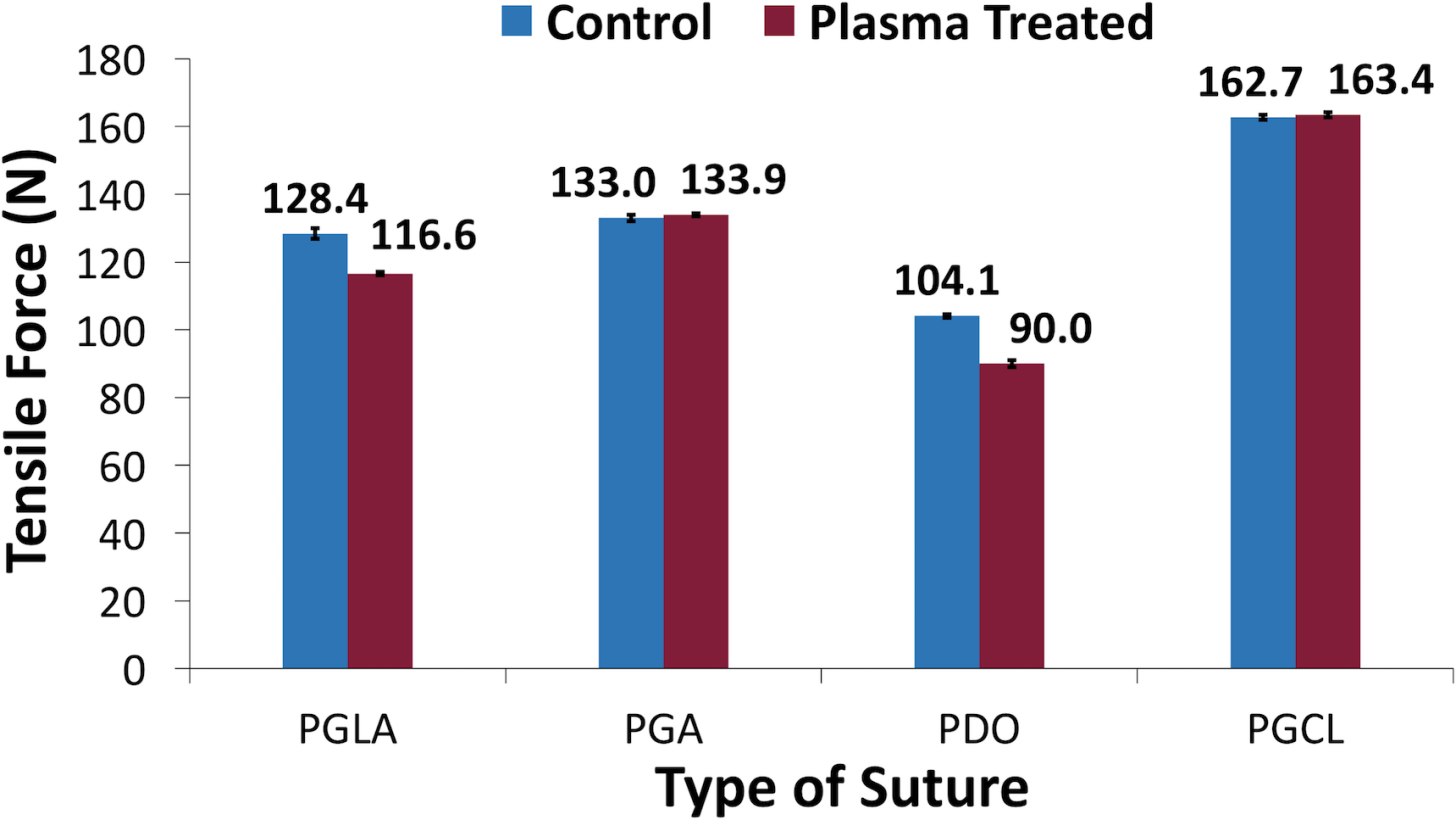
Maximum tensile force that sutures could withstand before and after 7-minute NTAP treatment. The maximum tensile force that PGA and PGCL sutures could withstand wasn’t altered by 7-minute NTAP treatment. However, 7-minute NTAP treatment led a decrease of the maximum tensile force that PGLA and PDO sutures could withstand.

As represented in Fig 7, after the 7-minute NTAP treatment of sutures, no statistically significant change on the maximum force that sutures can withstand was observed for PGA and PGCL. In detail, the maximum tensile forces, that untreated control and NTAP treated sutures can withstand, were measured as 133 N and 133.9 N for PGA (p> 0.05) and 162 N and 163 N for PGCL (p> 0.05), respectively. Conversely, the NTAP treatment significantly reduced the maximum force that PGLA and PDO sutures can withstand. The maximum tensile forces that untreated control and NTAP treated sutures can withstand were measured as 128 N and 116 N for PGLA (p< 0.05) and 104 N and 90 N for PDO (p< 0.05), respectively.

### In vitro scratch wound assay

**Fig 8:**
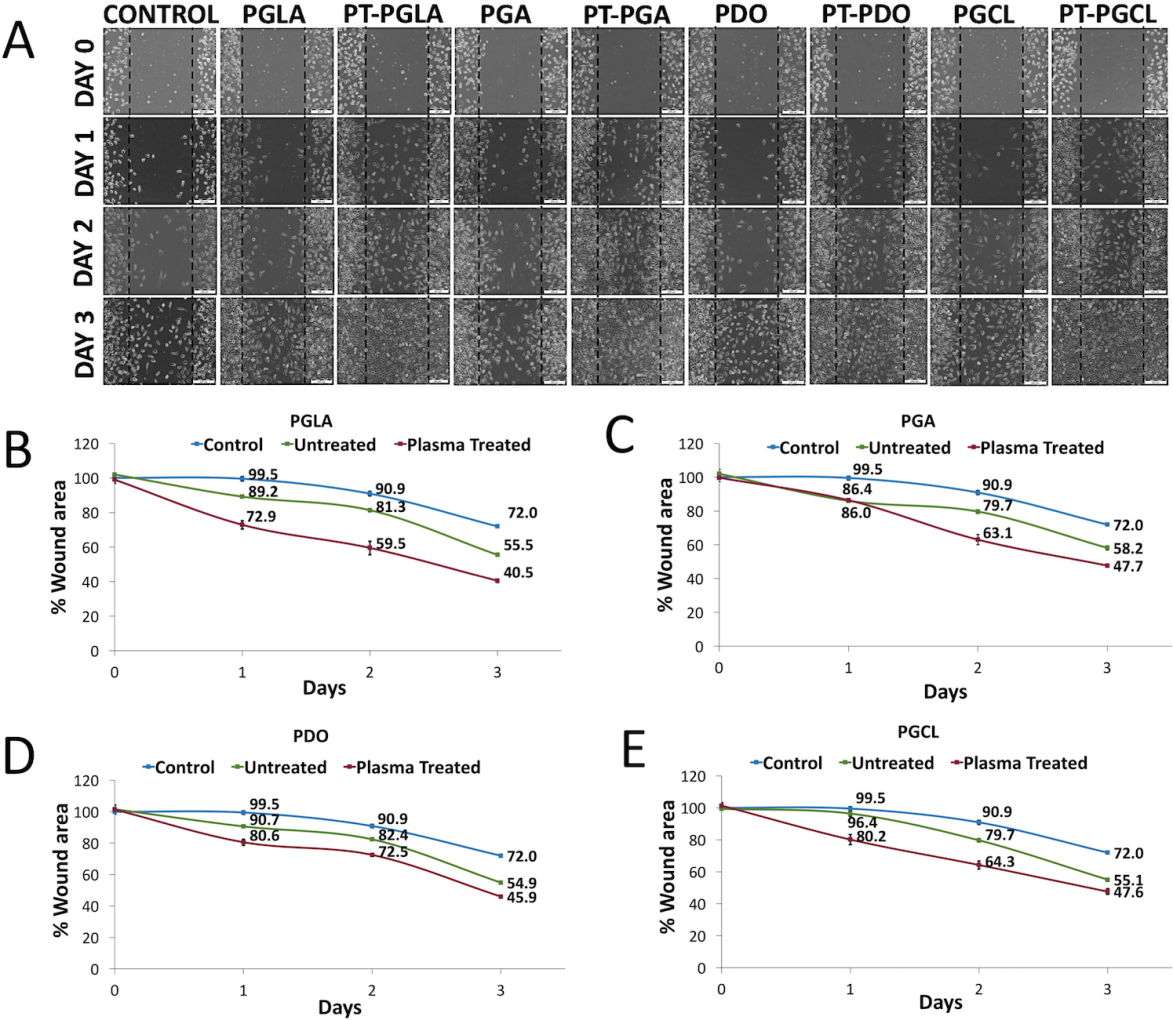
Effect of NTAP treated sutures on wound healing. (A) Light microscope images clearly show accelerated wound healing by introduction of 7-minute NTAP treated suture samples. Remaining wound areas when (B) PGLA, (C) PGA, (D) PDO and (E) PGCL sutures incubated on wound scratch model were quantified using ImageJ software.

As demonstrated in Fig 8A, all types of the 7-minute NTAP-treated suture samples evidently accelerate the healing of scratched wound area, in comparison to control and untreated suture samples after 3 days of creating the scratched wound. By the end of the 3 days after creating a scratch, the wound area was determined as 72% in the control group, while it dropped to 40.5%, 47.7%, 45.9% and 47.6% in NTAP-treated PGLA, PGA, PDO and PGCL sutures, respectively (p< 0.05) (Fig 8B–8E).

### Scanning electron microscopy (SEM) imaging

**Fig 9:**
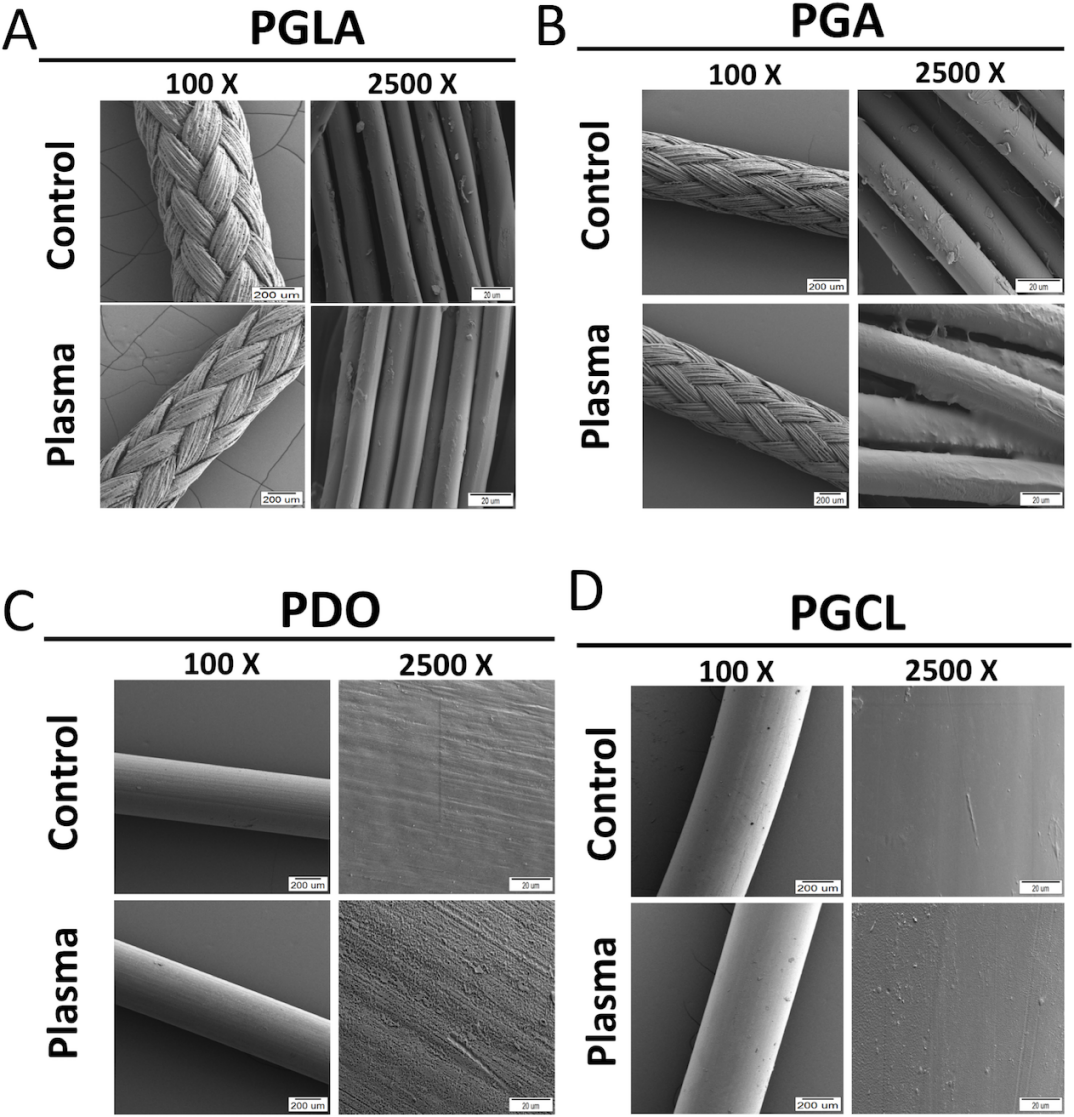
Scanning electron microscopy images of suture samples before and after NTAP treatment. SEM images of (A) PGLA, (B) PGA, (C) PDO and (D) PGCL sutures were obtained before and after 7-minute NTAP treatment at 100X and 2500X magnifications to evaluate structural and surface characteristics, respectively.

SEM micrographs of untreated control and the 7-minute NTAP-treated suture samples were collected at 100 X and 2500 X magnifications to observe any possible destructive effect on the suture materials and to evaluate modifications on suture the samples, respectively. During SEM imaging, the whole sample was scanned and representative images were presented in Fig 9A–9D. The average diameters of the sutures were about 0.5 μm. Multifilament PGLA and PGA sutures contain single filaments of 10–15 nm. 100 X magnified SEM micrographs did not reveal any destructive effect of NTAP treatment on all types of suture samples. However, the increased surface roughness in the NTAP-treated sutures, except PGLA, was observed in comparison to the control samples in 2500 X magnified SEM micrographs.

### Fourier transform infrared (FTIR) spectroscopy analysis

**Fig 10:**
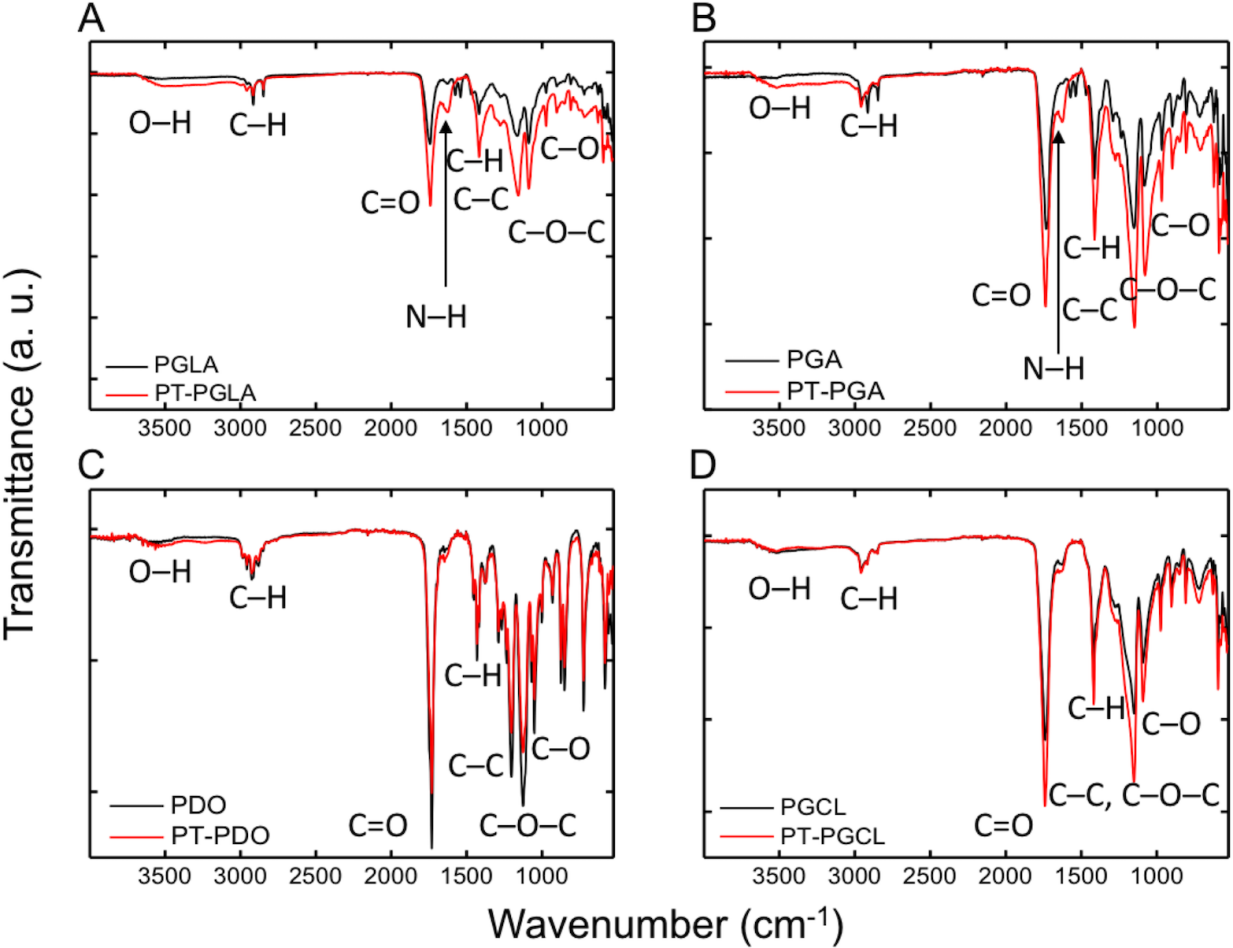
FTIR spectra of sutures before and after NTAP treatment. Chemical modifications caused on (A) PGLA, (B) PGA, (C) PDO and (D) PGCL sutures by 7-minute NTAP treatment was evaluated by using FTIR. FTIR spectra revealed that, 7-minute NTAP treatment, caused a decrease in relative absorption intensity of C-C groups and increase in relative absorption intensity of C–O and C = O groups suggesting increasing hydrophilicity.

Fig 10 shows FTIR spectra of untreated and the NTAP-treated sutures. The characteristic absorption bands were observed at around 1720 cm^-1^ (C = O, carbonyl), 1124 cm^-1^ (C–O–C, ether), 1070 and 1050 cm^-1^ (C–O, ester). The C–H stretching and bending modes were found at 2910 and 1427 cm^-1^ respectively. The range from 800–1200 cm^-1^ belongs to C-C stretches. Although there is no significant difference between the untreated and 7-minute NTAP treated PDO and PGCL sutures in the FTIR analysis, it is thought that the significant increase in the 1500–1700 cm^-1^ in the PGA and PGLA samples was attributed to the N-H groups. Similarly, the obvious broad bands of the PGA and PGLA samples in the range of 3100–3700 cm^-1^ were related to the increased–OH signals. In general, in this case of relative absorption intensity, C–O and C = O groups were increased while C–C groups were decreased upon the NTAP treatment.

### X-ray photoelectron spectroscopy (XPS) analysis

**Fig 11:**
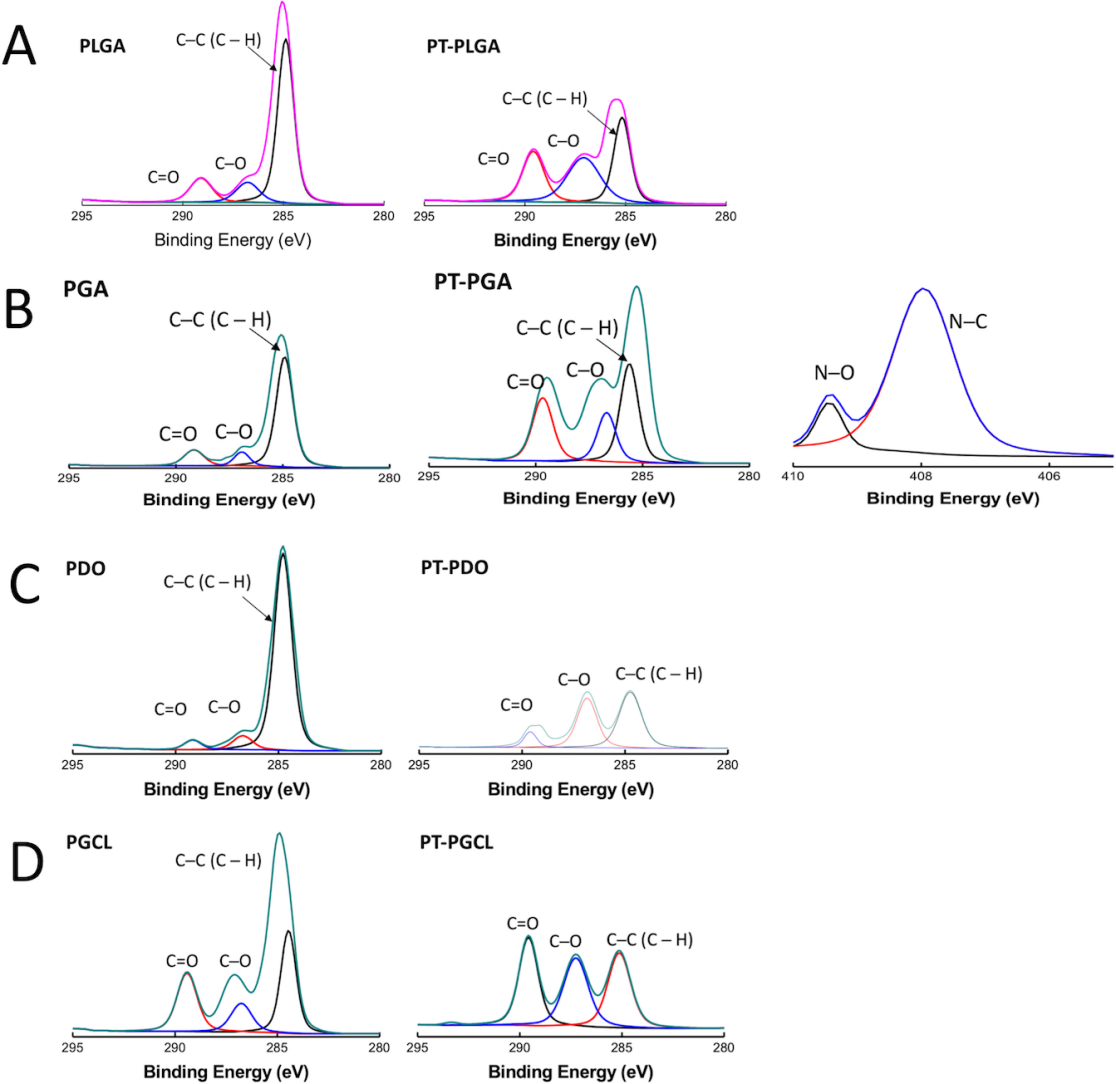
XPS C1s spectra of sutures before and after NTAP treatment. Chemical modifications caused on (A) PGLA, (B) PGA, (C) PDO and (D) PGCL sutures by 7-minute NTAP treatment was evaluated by using XPS. XPS spectra of C 1s revealed an increase in oxygen signals upon 7-minute NTAP treatment.

NTAP modification of the suture samples was also determined via XPS (Fig 11). The wide survey of the samples contained signals arising from the polymer backbone, namely C and O. No N component was noticeable; however, the N was detected for PGA after the NTAP treatment. The corresponding surface elemental compositions are given in Table 1. The oxygen signals increased in height due the increment in C–O and C = O bonds after the NTAP treatment. The photoelectron profiles of C 1s for the sutures before and after the plasma treatment show three distinct signals that are observed at around 285 eV, 287 eV, and 289 eV. These features are attributed to three different C environments in the structure, namely C–C, C–O, and C = O, respectively.

**Table 1:** Atomic composition and relative peak intensities of suture before and After 7-minute NTAP treatment.

Sutures		Atomic Composition (%)			Relative Peak Intensity (%)		
		C	O	N	C–C	C–O	C = O
**PGLA**	**Control**	81.90	18.10	0	78.74	9.68	11.57
	**NTAP treated**	65.55	34.44	0	47.47	24.69	27.84
**PGA**	**Control**	82.45	17.55	0	78.66	10.00	11.34
	**NTAP treated**	63.15	35.23	1.6	46.71	23.34	29.95
**PDO**	**Control**	70.40	21.04	0	89.33	6.33	4.34
	**NTAP treated**	40.98	49.30	0	46.18	40.92	12.90
**PGCL**	**Control**	67.97	27.66	0	54.19	15.09	30.72
	**NTAP treated**	52.54	44.63	0	32.29	29.65	38.06

## Discussion

Similar to all medical devices that are implanted to the human body, sutures provide an excellent medium for bacterial colonization. The presence of suture material on the surgical site significantly increases the susceptibility of the wound to infections. Suture-related surgical site infections are mostly linked to biofilm formation on the suture material [10].

Bacterial adhesion to the suture material is the crucial step for biofilm formation which might then lead to surgical site infections. The first 4 to 6 hours following the implantation of suture material to the surgical site has been reported as the most critical period for initial bacterial adhesion [39]. Moreover, adherence of bacteria on the suture surface, impairs the local mechanisms of decontamination of wound that is mediated by granulocytes [3]. Therefore, prevention of bacterial adhesion and/or eradication of adhered bacteria to suture during this period is important for the prevention of surgical site infections [5, 39].

Antimicrobial (such as triclosan) loaded sutures were developed to prevent surgical site infections. Despite the successful outcomes of triclosan-coated sutures *in vitro* and in clinical applications, its efficacy may remain insufficient in some surgeries [1]. Moreover, the potential toxic effects of triclosan on the reproductive system in humans and various animal models have been reported [10]. Triclosan also leads to the increased activity of efflux pump system, which than might cause promotion of multidrug resistance [40]. Therefore, we have attempted to utilize the NTAP treatment in order to develop novel antibacterial sutures for the prevention and control of surgical site infections.

The NTAP treatment induces strong and broad range of antimicrobial activity on various surfaces and its application for the decontamination of medical devices including cardiac pacemakers, metallic implants, and ultrasound contrast agents has been reported [28, 41, 42].

The antibacterial effect of the NTAP treatment was attributed to the synergistic effect of plasma-generated ROS including superoxide (O_2_^-^), hydrogen peroxide (H_2_O_2_), hydroxyl radical (OH^•^), ozone (O_3_) and RNS including nitric oxide (NO), nitrate (NO_3_^-^), nitrite (NO_2_^-^), peroxynitrite (ONOO^-^), which induce oxidative stress and subsequent oxidative damage such as membrane lipid peroxidation and oxidative damage of nucleic acids on microorganisms, and consequently cause inactivation [31, 41, 42]. Moreover, even though UV that was generated during the formation of NTAP is not considered as the dominant factor for antimicrobial activity, it might also contribute to the antimicrobial effect of the NTAP treatment [43].

In the present study, 3-minute NTAP treatment of all the tested contaminated suture materials resulted as complete inactivation of *E*. *coli* and *S*. *aureus* (Fig 2). Thus, NTAP treatment of the implanted suture immediately after the implantation could be considered as an effective method for eradication of adhered pathogens on sutures and prevention of surgical site infections.

In addition to the direct eradication of adhered bacteria on sutures by NTAP treatment, sutures could be modified to prevent bacterial colonization on them via relatively long NTAP treatment. As depicted in Fig 3, pre-treatment of sutures with NTAP for 7 minutes prevented bacterial colonization on sutures. It is important to note that the 7-minute NTAP-treated sutures provide a superior effect for the prevention of bacterial colonization on sutures compared to the antimicrobial loaded, (namely chlorhexidine diacetate) suture. Pre-treatment of sutures for 7 minutes with NTAP, prevented about 3-log of bacterial adhesion. This effect was uniform among the all tested suture materials including mono- and multifilament ones for both *S*. *aureus* and *E*. *coli*. In the present study, the prevention of the persistent bacterial colonization effect of the 7-minute NTAP treatment up to 10 days was also evaluated. As demonstrated in Fig 4A on the 10^th^ day after the 7-minute NTAP pre-treatment, bacterial growth around PGLA and PGA sutures were observed, whereas no growth was observed around PDO and PGCL sutures for *S*. *aureus* and *E*. *coli*. Such a result could be attributed to the differences in total surface area of mono- and multifilament suture materials. The differences in surface area of mono- and multifilament suture materials could be observed on the SEM images shown in Fig 9.

Monofilament sutures are manufactured as a single fiber, whose cross-section is circular. However, multifilament (or braided) sutures are produced by twisting relatively thin filaments which create a cross-section similar to the serrated circle with a larger total surface area [3, 44]. The unit surface area that comes in contact with the NTAP discharge (or in detail the ratio of the discharge to surface area) would be higher in monofilament sutures in comparison to multifilament sutures. Moreover, larger surface area and serrated surface of multifilament sutures constitute a complex three-dimensional surface topography which provides more adequate surface for bacterial adhesion [3]. Additionally, multifilament sutures were reported to be more susceptible for microbial colonization compared to monofilament sutures [3, 15]. Thus, within 7-minutes of the NTAP treatment period, multifilament sutures would be modified to a larger extent, in comparison to multifilament sutures.

With the close inspection of Fig 4A, one can note that the growth of *E*. *coli* around PGLA and PGA sutures were relatively less than the growth of *S*. *aureus* on the 10^th^ day after the 7-minute NTAP pre-treatment. This observation was found to be consistent with the data from the colony counting data. As shown in the Fig 4C, the number of *E*. *coli* on PGLA and PGA suture materials increases with the increasing delay time. However, the bacterial adhesion prevention effect of the NTAP treatment was not persistent for *S*. *aureus* on PGLA and PGA suture materials. Even 1 day after the 7-minute NTAP treatment of PGLA and PGA sutures, about 3-log growth per cm of the suture fragment for *S*. *aureus* that is similar to control group was observed. Previous studies reported that *S*. *aureus* is more prone to adhere especially on multifilament suture surfaces compared to *E*. *coli*. Moreover, *S*. *aureus* tend to adhere suture surfaces in clusters whereas *E*. *coli* adhere suture surfaces individually [2, 45]. Suture material by itself could also be a factor for bacterial adhesion. Masini et al. reported that PGA sutures lead to higher bacterial adherence compared to other suture materials [20]. Furthermore, Grigoras et al. reported that PGA suture material allow the highest bacterial adherence whereas the PDO sutures allowed the least bacterial adherence [9].

Antibacterial, bacterial adhesion and growth preventive effects of NTAP-treated various surfaces such as latex, alginate gel and metals have been reported in the literature [35, 37, 38]. Accumulation of plasma-generated ROS, RNS and free radicals, such as the aforementioned superoxide (O_2_^-^), hydrogen peroxide (H_2_O_2_), hydroxyl radical (OH^•^), ozone (O_3_) nitric oxide (NO), nitrate (NO_3_^-^), nitrite (NO_2_^-^), peroxynitrite (ONOO^-^) on material surfaces and chemical modification of the NTAP-treated surfaces with those plasma generated ROS, RNS and free radicals are thought to be the source of antimicrobial and bacterial adherence preventive effect of plasma [35, 38].

Despite the lack of a general agreement regarding the surface hydrophobicity/hydrophilicity and bacterial adherence, hydrophobic interactions in between the surface and bacteria is considered to contribute to bacterial adhesion to surface and subsequent biofilm formation. For instance, several studies have shown that bacterial adherence and subsequent bacterial colonization and biofilm formation could substantially be reduced on hydrophilic surfaces [9, 37, 46]. Moreover, UV generated during the formation of NTAP might also contribute to the prevention of bacterial adhesion to the suture surface as increased hydrophilicity on photofunctionalized titanium surfaces was reported previously [46]. As shown in Fig 5A, the contact angle of all tested suture materials decreased substantially and surfaces became superhydrophilic after the 7-minute NTAP treatment. The superhydrophilic character of the suture material could be the primary factor for the prevention of bacterial colonization on NTAP treated sutures. The increased hydrophilicity of suture materials after the NTAP treatment could be linked to air-plasma generated active species such as atomic oxygen (O), hydrogen peroxide (H_2_O_2_), hydroxyl radical (OH^•^), ozone (O_3_), and free radicals which have possible reactivity with suture materials to form polar groups on the surface of the sutures. In the present study, oxygen containing groups such as carboxylic acid (COOH) might have been introduced on the suture surfaces since the presence of both–OH and C–O, and C = O bonds were confirmed by the FTIR analysis. In addition, changes in the atomic compositions, particularly, the intensity of C–C, C–O, and C = O bonds upon the NTAP treatment when compared to that of non-treated sutures have been demonstrated by XPS the analysis.

One of the primary functions of sutures is to provide mechanical support during the healing process of surgical wounds [8, 20]. Therefore, it is important to rule out any possible negative effects of the NTAP treatment on the tensile strength of sutures. The maximum tensile force that sutures could withstand were determined before and after the 7-minute NTAP treatment. No statistically significant changes in the maximum tensile strength of PGA and PGCL sutures were found. The maximum tensile strength of PGLA and PDO significantly decreased as depicted in Fig 7. However, the reduction of the tensile strength of PGLA and PDO sutures following the 7-minute NTAP treatment was not considered a drawback in terms of possible practical application based on European Pharmacopoeia. The minimum tensile strength value of sutures required by European Pharmacopoeia is reported to be 50.8 N [47]. Overall, the maximum tensile strength for all sutures including those that are NTAP-treated, were within the limits required by European Pharmacopoeia.

In addition to the antimicrobial effect of NTAP and NTAP treated materials, the wound healing effect of NTAP and NTAP treated materials has been demonstrated [48–50]. Taken together, NTAP treatment has selective effects, in which, it provides microbial inactivation while promoting the growth of eukaryotic cells [51]. In the present study, no adverse effect of NTAP-treated suture materials on the viability of L929 cell line was observed. On the contrary, the promotion of growth of L929 cells when incubated with the 7-minute NTAP-treated suture materials were observed. As demonstrated in Fig 8, the visual inspection of light microscopic images of scratch wound areas revealed that, the 7-minute NTAP-treated suture materials remarkably increased the wound healing when compared to control and untreated-suture groups for all time points. Increase in wound healing was more prominent on the 3^rd^ day of incubation when most of the wound area was covered with L929 cells. Quantification of wound healing has shown that even untreated sutures have shown a promoted wound healing effect compared to the control group. Beyond that, the 7-minute NTAP-treated sutures have significantly increased the wound healing process. On day 3, around 55%–60% of the wound area was covered with L929 cells, depending on the suture material, while the closed wound area was determined as 28% and about 40–45% for control and untreated suture groups, respectively (Fig 8B–8E). The wound area, determined for various types of the 7-minute NTAP-treated sutures, didn’t show any statistically significant difference. As previously indicated in the literature, reactive oxygen species (ROS) generated with NTAP act as a signal molecule and accelerate the wound closure [52–55]. In another study, Murrell et al. reported that ROS significantly promotes the migration and proliferation of human fibroblasts [56]. In addition, we previously demonstrated that the NTAP-treated electrospun polyvinyl alcohol (PVA)/polyacrylic acid (PAA) nanofibers significantly increased the wound closure when extracted media was applied on the scratch assay developed by L929 mouse fibroblasts [57]. Therefore, we strongly speculated that extract media acts as an ROS transfer media to the scratch site and fastens wound closure by enhancing the fibroblast migration and proliferation.

As absorbable sutures were tested in the present study, the degradability of the 7-minute NTAP-treated sutures were also evaluated along with untreated ones. Biodegradability of the polymeric sutures is defined as the breakdown of the molecular bonds by enzymatic or hydrolytic processes in physiological environment [58, 59]. In particular, PGA, PLGA, PDO and PGCL based sutures are mainly degraded by the hydrolytic mechanism where water molecules penetrate and cleavage the ester linkages [60]. The protons on the carboxyl end groups of degradable sutures are considered as the driving force of this degradation process [59]. The temperature, ester group density, surface area, molecular weight and structure are the major factors that influence hydrolytic biodegradation [44]. In this study, *in vitro* degradation behaviour of different sutures was analyzed by incubating in PBS at 37°C and pH 7.4 as previously reported by Im et al. [61]. The weights of control and 7-minute NTAP-treated PGA and PGCL sutures didn’t display statistically significant difference (p> 0.05), whereas the weight difference of control and the 7-minute NTAP-treated PGLA and PDO sutures were found to be statistically significant on the last weight measurement where the suture fragments were shattered during the manipulation for measurements. Such change could be correlated to the tensile force measurement experiments in which statistically significant reduction in the maximum tensile force that sutures can withstand was found for 7-minute NTAP-treated PGLA and PDO sutures while no statistically significant change was determined for PGA and PGCL sutures. Despite the alterations on the degradation patterns of 7-minute NTAP treated sutures when compared to the control group, the time points in which the suture materials got shattered haven’t changed. These results could be explained by the extent of chemical the modification exerted by NTAP treatment, which is limited to the surface of suture rather than to penetrate to bulk structure as the degradation process starts from the surface of polymers and proceeds step by step towards the deeper layers [44].

The wound support period of PGLA, PGA, PDO and PGCL sutures in physiological conditions were reported as approximately 30, 30, 60 and 21 days, respectively by the manufacturer. Our data shows that the 7-minute NTAP treatment didn't affect the wound support period which is the time that the suture material can withstand the tensile forces exerted by the open wound and the duration for which the suture can provide mechanical support to wound. Overall, on the basis of tensile force measurement and degradation experiments, the 7-minute NTAP treatment hasn't significantly affected the mechanical and wound support properties of all tested sutures.

## Conclusions

In the present study, the efficacy of the NTAP treatment on the inactivation of *S*. *aureus* and *E*. *coli* and the prevention of *S*. *aureus* and *E*. *coli* colonization on the NTAP treated-sutures were evaluated. We have shown that the 3-minute NTAP treatment could lead to complete inactivation of 3-log/cm suture of *S*. *aureus and E*. *coli*. Furthermore, the NTAP pre-treatment of sutures prevented the colonization of *S*. *aureus and E*. *coli* on sutures. The colonization preventive effect of NTAP pre-treatment lasted up to 10 days for PDO and PGCL. NTAP-treated sutures also contributed and accelerated the wound healing in a scratch wound model. Mechanical properties of sutures were also evaluated and our results indicated that the NTAP treatment didn’t affect mechanical properties of sutures adversely. Chemical characterization results revealed that the plasma generated ROS/RNS interacted with the suture material and made the surface of sutures more hydrophilic which, seems to central to NTAP treatment mediated effects. To the best of our knowledge the present study is the first in the literature reports to prove that NTAP treatment of surgical sutures remarkably prevents bacterial adhesion for the prevention of suture related-SSIs.

In conclusion, NTAP treatment of PGLA, PGA, PDO and PGCL sutures effectively inactivate and prevent bacterial growth on surgical sutures, and NTAP-treated sutures could be considered as a possible novel alternative for the prevention and control of suture-related surgical site infections.

## Supporting information

S1 Fig Results of a pilot study to determine the NTAP time for decontamination experiments. Pilot experiment to determine the required NTAP treatment time for decontamination was carried out on PGLA sutures with *E*. *coli*. Number of surviving bacteria on sutures after NTAP treatment decreased with increasing treatment time. 3-minute NTAP treatment led to complete inactivation of bacteria.
(PDF)

S2 Fig Results of a pilot study to determine the NTAP time for prevention of bacterial colonization on sutures. Pilot experiment to determine the required NTAP treatment time for for prevention of bacterial adhesion was carried out on PGLA sutures with *E*. *coli*. Number of surviving bacteria that adheres on sutures after NTAP treatment decreased with increasing treatment time. After 7-minute NTAP treatement no bacterial adhesion was observed.
(PDF)

S3 Fig XPS survey spectra of sutures. XPS survey spectra of (A) PGLA, (B) PGA, (C) PDO and (D) PGCL before and after 7-minute NTAP treatment.
(PDF)
