# Supplementary material for: Prevention of bacterial colonization on non-thermal atmospheric plasma treated surgical sutures for control and prevention of surgical site infections
Source: PLoS One. 2018 Sep 5;13(9):e0202703. doi: 10.1371/journal.pone.0202703 (PMC6124751; doi:10.1371/journal.pone.0202703)
Supplement: S1 Fig — Pilot experiment to determine the required NTAP treatment time for decontamination was carried out on PGLA sutures with E. coli. Number of surviving bacteria on sutures after NTAP treatment decreased with increasing treatment time. 3-minute NTAP treatment led to complete inactivation of bacteria. (PDF) [file pone.0202703.s001.pdf]

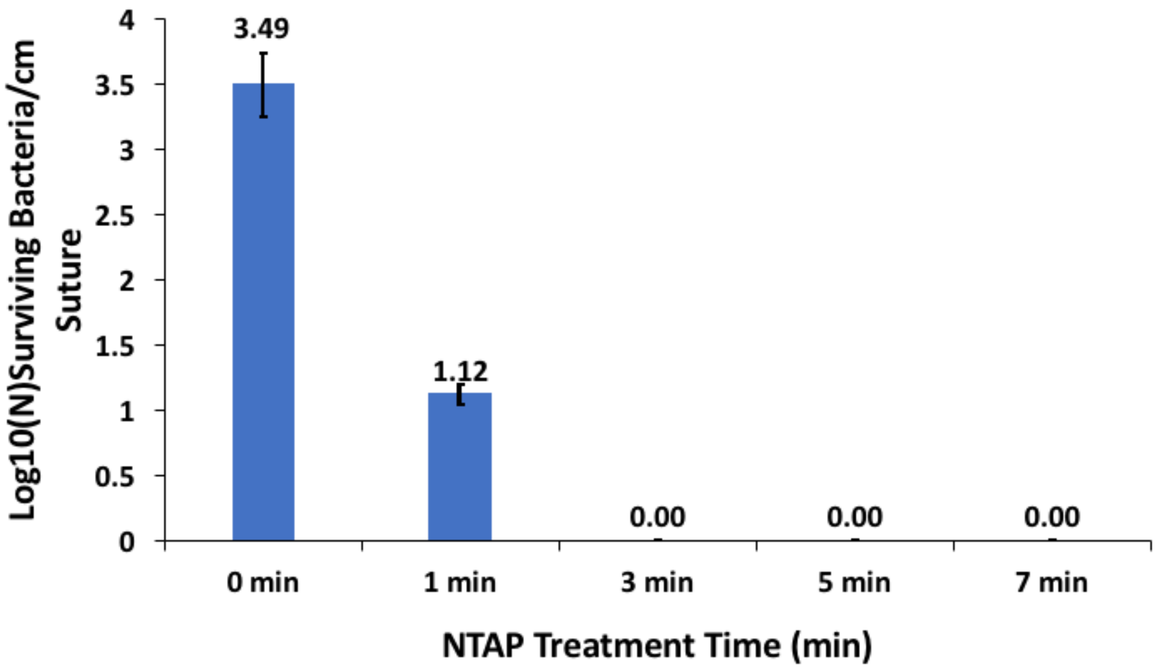

S1 Fig. Results of a pilot study to determine the NTAP time for decontamination experiments. Pilot experiment to determine the required NTAP treatment time for decontamination was carried out on PGLA sutures with *E. coli*. Number of surviving bacteria on sutures after NTAP treatment decreased with increasing treatment time. 3-minute NTAP treatment led to complete inactivation of bacteria.
