# Supplementary material for: Prevention of bacterial colonization on non-thermal atmospheric plasma treated surgical sutures for control and prevention of surgical site infections
Source: PLoS One. 2018 Sep 5;13(9):e0202703. doi: 10.1371/journal.pone.0202703 (PMC6124751; doi:10.1371/journal.pone.0202703)
Supplement: S2 Fig — Pilot experiment to determine the required NTAP treatment time for for prevention of bacterial adhesion was carried out on PGLA sutures with E. coli. Number of surviving bacteria that adheres on sutures after NTAP treatment decreased with increasing treatment time. After 7-minute NTAP treatement no bacterial adhesion was observed. (PDF) [file pone.0202703.s002.pdf]

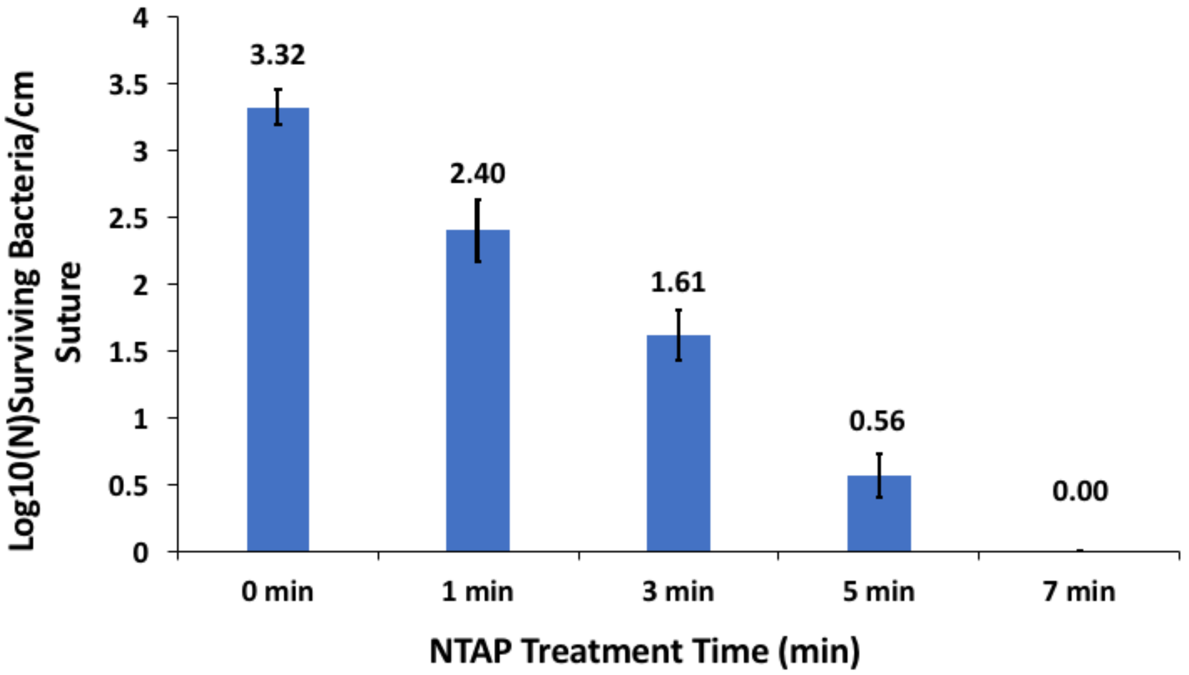

S2 Fig. Results of a pilot study to determine the NTAP time for prevention of bacterial colonization on sutures. Pilot experiment to determine the required NTAP treatment time for prevention of bacterial colonization was carried out on PGLA sutures with *E. coli*. Number of surviving bacteria that colonized on sutures after NTAP treatment decreased with increasing treatment time. After 7-minute NTAP treatement no bacterial colonization was observed.
