# Supplementary material for: Prevention of bacterial colonization on non-thermal atmospheric plasma treated surgical sutures for control and prevention of surgical site infections
Source: PLoS One. 2018 Sep 5;13(9):e0202703. doi: 10.1371/journal.pone.0202703 (PMC6124751; doi:10.1371/journal.pone.0202703)
Supplement: S3 Fig — XPS survey spectra of (A) PGLA, (B) PGA, (C) PDO and (D) PGCL before and after 7-minute NTAP treatment. (PDF) [file pone.0202703.s003.pdf]

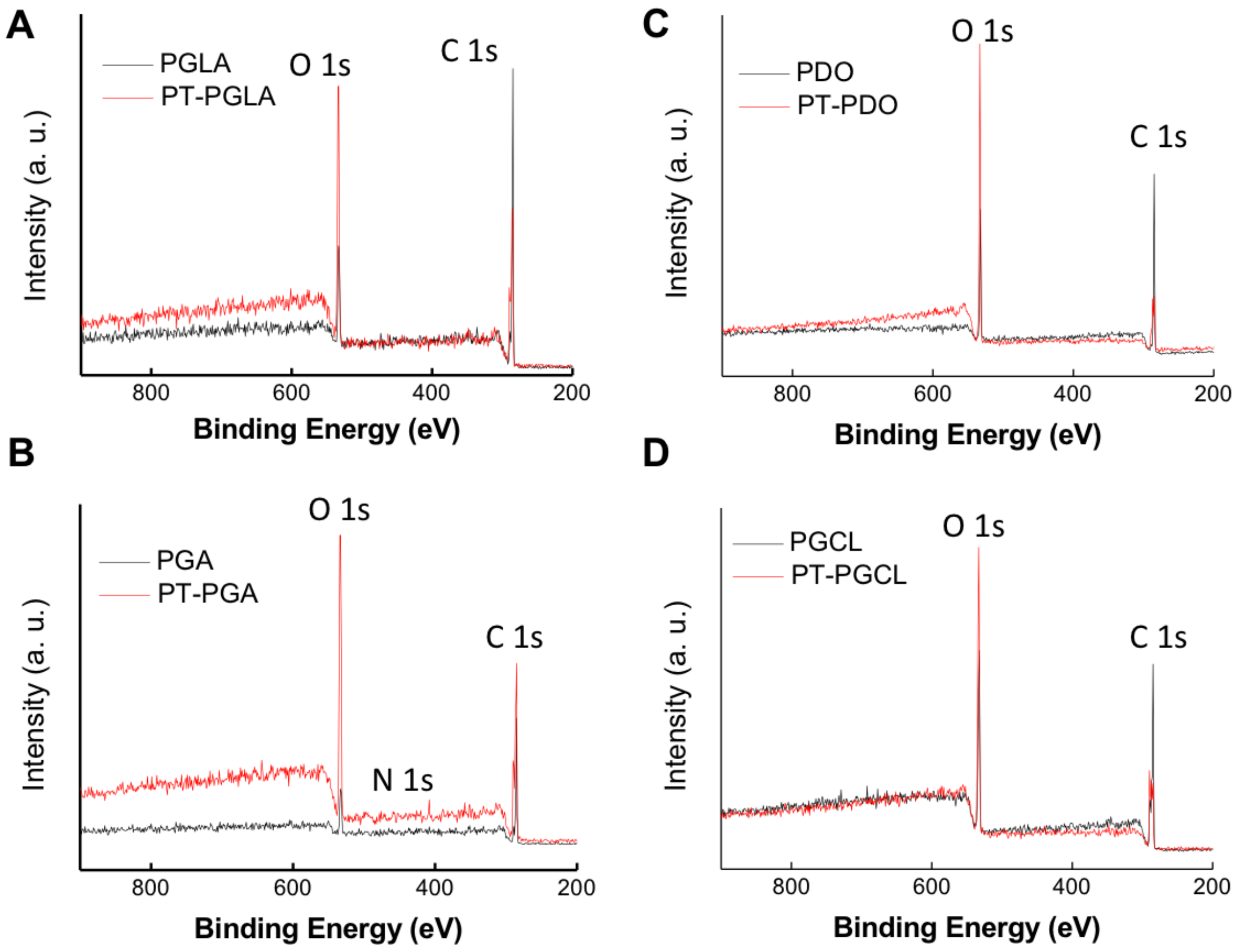

S3 Fig. XPS survey spectra of sutures. XPS survey spectra of (A) PGLA, (B) PGA, (C) PDO and (D) PGCL before and after 7-minute NTAP treatment.
